# Barriers to gender-equitable HIV testing: going beyond routine screening for pregnant women in Nova Scotia, Canada

**DOI:** 10.1186/1475-9276-10-18

**Published:** 2011-05-11

**Authors:** Jacqueline C Gahagan, Janice L Fuller, E Michelle Proctor-Simms, Todd F Hatchette, Larry N Baxter

**Affiliations:** 1 Gender and Health Promotion Studies Unit, School of Health and Human Performance, Dalhousie University, Halifax, Nova Scotia, Canada; 2 Nova Scotia Advisory Commission on AIDS, Halifax, Nova Scotia, Canada; 3 Queen Elizabeth II Health Science Centre, Division of Microbiology, Department of Pathology and Laboratory Medicine, Halifax, Nova Scotia, Canada

## Abstract

**Background:**

Women and men face different gender-based health inequities in relation to HIV, including HIV testing as well as different challenges in accessing HIV care, treatment and support programs and services when testing HIV-positive. In this article, we discuss the findings of a mixed methods study exploring the various individual and structural barriers and facilitators to HIV counselling and testing experienced among a sample of adult women and men living in Nova Scotia, Canada.

**Methods:**

Drawing from testing demographics, qualitative interview data and a review of existing testing policies and research, this paper focuses on understanding the gendered health inequities and their implications for HIV testing rates and behaviours in Nova Scotia.

**Results:**

The findings of this research serve as the basis to further our understanding of gender as a key determinant of health in relation to HIV testing. Recognizing gender as a key determinant of health in terms of both vulnerability to HIV and access to testing, this paper explores how gender intersects with health equity issues such as access to HIV testing, stigma and discrimination, and sexual behaviours and relationships.

**Conclusions:**

Drawing on the current gender and HIV literatures, in conjunction with our data, we argue that an enhanced, gender-based, context-dependent approach to HIV counselling and testing service provision is required in order to address the health equity needs of diverse groups of women and men living in various settings. Further, we argue that enhanced HIV testing efforts must be inclusive of both men and women, addressing uniquely gendered barriers to accessing HIV counselling and testing services and in the process moving beyond routine HIV testing for pregnant women.

## Introduction

HIV infection remains a significant public health issue, particularly in terms of the sexual and reproductive health equity and health outcomes of many Canadians. In 2008 it was reported that there were between 2300 and 4300 new HIV infections in Canada, bringing the estimated total number of people living with HIV in Canada to 65000 [1]. It is estimated that 26% of Canadians living with HIV are unaware of their infection since they have not been tested, suggesting that approximately 16900 Canadians are HIV positive yet unaware of their HIV status [1]. While men who have sex with men continue to make up the majority of new HIV cases, the number of new infections in Canada due to heterosexual activity with an infected partner has more than doubled since the late 1980s [2]. In addition, there has been a steady increase in the proportion of positive HIV tests amongst women, with female cases accounting for 26% of reported adult HIV infections in 2008 [3]. HIV infection in Canada is also becoming more prevalent among other populations who face considerable health equity issues such as Aboriginal populations, persons from countries where HIV is endemic, individuals in correctional facilities, those with lower socio-economic status, and the homeless [4,5].

Gender, as a key determinant of health, has specific relevance to HIV infection and related health equity issues in terms of how gender influences the activities of everyday life for both men and women. Unlike biological sex, gender refers to the socially constructed notion of what are considered appropriate roles and behaviours for men and women in society [6]. Distinct gender roles and behaviours for men and women can lead to inequities in both health status and access to health care [6,7]. Gender-based role expectations and associated power dynamics can influence men's and women's sexual behaviours and contexts of HIV risk, creating a variety of challenges in avoiding HIV infection, as well as the likelihood of help-seeking behaviours for treatment and necessary care once infected [8]. Gender is widely recognized as a key factor in terms of vulnerability to HIV infection, access to testing, care and treatment, and yet how to effectively address the gendered health inequities and social consequences associated with HIV infection remains less clear [9].

In terms of HIV testing, it is suggested that women and men face different gender-related inequities to testing as well as different challenges in accessing HIV care, treatment and support programs and services when testing HIV-positive [10,11]. It is therefore noteworthy that the leading cause of death among women of reproductive age globally is HIV/AIDS [12]. Clearly, gender-based inequities are related to challenges in negotiating safer sex, including barrier methods such as the use of condoms, which often render women and girls particularly vulnerable to HIV infection. Social and economic inequities, such as poverty and marginalization, further contribute to the gendered vulnerability of women and girls to HIV infection both in Canada as well as globally [2,4,13]. From a population health perspective women, whether from the global north or south, constitute a diverse social group, with multiple, intersecting identities and roles that influence their experiences of the world and specifically, in relation to HIV/AIDS prevention, care, treatment and support [14,15].

Men as a population also experience distinct gender-based HIV risk contexts and barriers to HIV testing. For example, masculinity has its own set of implications in relation to fuelling the HIV/AIDS epidemic, including gendered sexual behaviour-related expectations and attitudes that may impact the sexual health and wellbeing of men and their sexual partners [10]. Masculine sexual norms often encourage men to engage in more risky sexual behaviours, including having multiple sexual partners and not using condoms [16-18]. Additionally, research has shown that heterosexual men often do not see HIV testing as their concern but rather regard this as the responsibility of their female sexual partners [19].

Gender as a social construct also influences HIV infection and related health equity for marginalized and vulnerable groups such as gay men, bisexual men, lesbians, bisexual women, transgender persons, and transsexuals. Social stigma and economic disadvantage experienced by such populations increase their vulnerability to HIV/AIDS due to marginalization, inequity, discrimination, criminalization, oppression and violence [20]. Thus, it is important to examine HIV counselling and testing strategies through a gender equity lens in order to acknowledge and address some of the complexities that influence a person's ability to seek out and make use of such health services [16].

In this article we discuss the gendered implications of findings from a mixed methods study on how HIV counselling and testing is experienced and perceived by a sample of adult men and women living in the east-coast province of Nova Scotia, Canada with a population of approximately 1 million. The specific objectives of this study were: 1) to understand the HIV testing rates and behaviours among individuals in Nova Scotia; and 2) to develop an evidence-based understanding of the experiences of individuals in relation to HIV testing in order to develop Nova Scotia-specific policy and programming recommendations. As an emergent outcome, we identified the significance of gender as a key determinant of health in HIV testing uptake rates and related testing experiences in Nova Scotia.

Although the international shifts in HIV testing policy and programming present an opportunity to re-examine current approaches, the issue of gender and related determinants of health equity remain largely absent from the debate. Based on the findings of this study, we argue for the need to further consider the gendered nature of HIV counselling and testing experiences and perceptions as a means of addressing the inequities in testing uptake rates, particularly among populations less likely to be tested. In light of the Public Health Agency of Canada (PHAC) forthcoming revisions of the Canadian Medical Association (CMA) HIV testing guidelines from 1995 and the US Centers of Disease Control (CDC) suggested changes to the provision of HIV testing, we regard this as a critical opportunity to specifically re-examine Nova Scotia's HIV testing approach. In other words, we regard this as a timely means of exploring gendered notions about HIV testing that includes, but goes beyond, pre-natal HIV testing in an effort to ensure current testing approaches are restructured to be more inclusive and equitable for all populations in Nova Scotia.

### HIV Counselling and Testing in Nova Scotia, Canada

Since the beginning of the epidemic, more than 700 persons have tested positive for HIV in Nova Scotia, though the actual number is likely higher since not all HIV positive individuals get tested, and not all HIV positive individuals living in Nova Scotia are tested in the province [1]. In Nova Scotia there are three types of HIV testing available: nominal testing (since 1985), non-nominal testing (since 1991), and anonymous testing (since 1994). There is currently no rapid testing routinely offered in the province. The anonymous HIV testing service in Nova Scotia was initiated in Halifax, the province's capital, and in 2007 it became available in Sydney, the second largest urban centre in Nova Scotia, with some outreach to rural communities in eastern Nova Scotia. Despite these efforts, there remain a number of significant equity issues to accessing HIV counselling and testing in Nova Scotia, including geographic isolation, a lack of anonymous testing sites, fear of disclosure in small communities, poverty, and the continuing stigma associated with HIV and HIV testing [21]. Further, there is limited evidence regarding the reasons for seeking or not seeking HIV counselling and testing services in Nova Scotia, and consequently there is limited information on how these services could be improved to meet the needs of diverse populations of men and women across the province. Without an effective continuum of primary and secondary HIV prevention, including a fuller understanding of HIV testing approaches and inequities, those in need of care, treatment and support may be missed by our public health efforts.

It is therefore crucial to understand HIV testing behaviours in Nova Scotia, considering that HIV counselling and testing can help prevent the further spread of HIV, and provides a means of facilitating timely access to care, treatment and support for those who are found to be HIV positive. Therefore the key objectives of this study focused on HIV testing rates and behaviours among males and females between the ages of 15 and 65 in Nova Scotia in order to develop a better understanding of the experiences of individuals that have or have not been tested for HIV within the last year. In this paper, we describe a number of the gendered implications of HIV counselling and testing in Nova Scotia, and potential ways of improving the HIV services offered to help address health equity issues for both women and men in diverse communities across the province.

## Methods

Both quantitative (HIV surveillance) and qualitative (in-depth interviews) data were collected for this mixed methods study, as well as a review of existing HIV testing policies and research. Ethics approval for this research study was granted by the Capital District Health Authority (CDHA) Ethics Review Board prior to the commencement of data collection. All data collected were treated as confidential by members of the research team, and each researcher was required to sign a confidentiality agreement to that effect. The progression of the research was guided with the assistance of a Community Advisory Committee, whose members contributed to the development of the interview guide, assisted with promoting the study, and provided feedback for publications and other knowledge translation and exchange activities.

The HIV surveillance data (including provincial updates of testing and information about positive tests) were gathered in collaboration with the Nova Scotia Department of Health Promotion and Protection (HPP) and the CDHA, and demographic information about anonymous HIV tests was provided by the Halifax Sexual Health Centre (HSHC). Data collected for all HIV screening tests performed at the CDHA laboratory between April 16^th ^2009 and April 15^th ^2010 were extracted from the Laboratory Information System. All cases are defined by having positive Western Blots as confirmation testing and all confirmatory tests are conducted by the CDHA laboratory in Halifax. Data fields analyzed included: age group, sex, probable prenatal screen and test setting. All data collected were anonymized and aggregated, and therefore impossible to link back to individuals who had been tested, and all copies of the surveillance data were used only for the purpose of this study. These data sets provided a snap shot of the HIV testing uptake rates for Nova Scotia and allowed for a better understanding of inequities in testing.

In addition, in-depth interviews were conducted with a total of 50 adults between the ages of 15 and 65 who had lived in Nova Scotia for at least the past year, and had either been tested in Nova Scotia or had not been tested for HIV in the past year (prior to the interview). Social inclusion and equity were priorities of this research, and as such representation from various populations and communities was sought, including: Aboriginal, African Nova Scotian, Caucasian and Immigrant populations, as well as urban and rural participants. Participants volunteered and self-selected to be a part of the study. Recruitment occurred through circulating flyers, and the study was promoted with the cooperation of both community and health care services contacts, including people living with HIV, health care providers, health researchers, public health policy experts, and community-based workers across the province. Eligibility criteria for inclusion into the study required the potential participants to be able to speak and understand English, to have lived in Nova Scotia at the time they were tested, and where applicable, to have been tested within the past year. Participants received a one-time, $15 honorarium to help compensate them for their time away from their other commitments and travel expenses. The in-depth, semi-structured interviews were conducted one-on-one in private meeting spaces or over the telephone with a member of the research team. Participants were given the opportunity to discuss the study and provided their informed consent prior to being interviewed about their experiences and perceptions of HIV counselling and testing in Nova Scotia. Paper resources with information about HIV and testing were offered to each participant at the conclusion of their interview.

The interviews were audio recorded with the consent of the participants, and then transcribed by a trained transcriptionist. The interview transcripts were then thematically analyzed with the assistance of qualitative data management software (Atlas ti). The iterative analysis process included reading and re-reading the transcripts, creating codes from a systematic review of the data, and allowing for the emergence of themes. Atlas ti was used to assist in coding and identifying frequent and unique issues to emerge from the qualitative data. The data were subjected to thematic analysis and categorized with attention to the common themes and with respect to the diverse perceptions and experiences of the participants.

It is important to note that the qualitative and quantitative data collected for this study had different analytic intentions in that the quantitative data were meant to provide a background to male and female testing rates while the qualitative data were meant to provide a greater understanding of the context, experiences and perceptions of HIV testing. In this paper we provide an overview of the emergent theme of gender and its implications for HIV testing with a focus on providing a rich description of individual experiences. We combine data sources in an effort to offer a more robust understanding of the testing gaps, potential barriers and facilitators which can prove useful in informing evidence-based HIV testing policy and programming initiatives in Nova Scotia.

## Results

The quantitative findings from the surveillance data examine the sex and age characteristics of HIV screening test recipients between April 16^th ^2009 and April 15^th ^2010. By using multiple methods of data collection and analysis, including an examination of the surveillance and interview data in conjunction with a review of previous research and testing policies, a more comprehensive picture of the experiences and perceptions of HIV testing and counselling in Nova Scotia emerged. The qualitative findings reported in this article focus on the gendered implications of HIV counselling and testing in Nova Scotia and focus on the themes that emerged from the perceptions and experiences of the interview participants, both from those who had and had not been tested in the past year (prior to the time of the interview). These three key themes include: perceptions of routine testing, stigma and discrimination, and sexual behaviours and relationships. What follows is a description of the quantitative and qualitative results and findings in greater detail, beginning with the quantitative surveillance data.

### Surveillance Data

The Nova Scotia laboratory data examined between April 16^th ^2009 and April 15^th ^2010 indicate that approximately equal numbers of males and females were tested for HIV, when prenatal HIV tests were excluded. The age distribution of HIV tests was also similar for males and females. Newly reported HIV cases were predominantly male and tended to have an older age distribution compared to the HIV screening test distribution. The most commonly reported HIV exposure type for newly diagnosed HIV cases was men who have had sex with men (MSM), while the majority of people who went for anonymous HIV tests did not have any identified risk (NIR) for HIV infection, a category within which a person who does not identify any other possible route of HIV transmission would be classified. Age and sex distribution of anonymous tests was similar to that for all tests at CDHA lab, and the anonymous testing service in Halifax were accessed mostly by Caucasian individuals aged 20-30, with near equal representation of male and female clients. At the time of writing, there was no rapid or point of care testing routinely offered in Nova Scotia.

#### A) HIV Screening Tests: Sex and Age Characteristics

**Figure 1:**
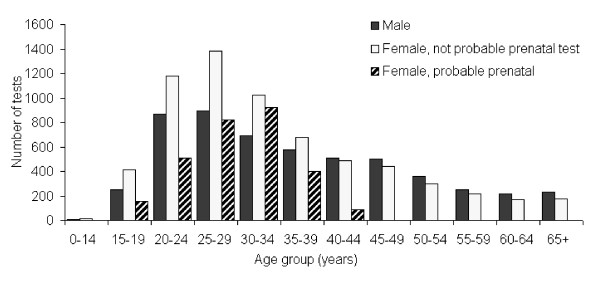
**Number of HIV Screening Tests by Sex and Age Group, Females Disaggregated by Probable Prenatal Test, CDHA Laboratory, April 16^th ^2009 - April 15^th ^2010**.

Of the 15,518 HIV screening tests performed by CDHA laboratory between April 16^th ^2009 and April 15^th ^2010, 95% had age and sex recorded (14,750). Anonymous tests account for the majority of tests for which age and sex information was unavailable. Figure 1 presents HIV screening tests by sex and age group, with females divided into two groups: probable prenatal HIV screening test and not probable prenatal HIV screening test.

Approximately 64% of HIV screening tests performed at the CDHA laboratory with known sex was for females. Although data regarding whether tests were submitted for prenatal screening is not routinely available from laboratory data, an estimate can be made by examining data from females aged 15 to 44 years whose specimen for the HIV screen was collected on the same day as specimens for both syphilis and rubella (this combination of tests would be unusual outside the context of prenatal screening). Using this surrogate estimate, a large proportion of female tests were likely conducted as part of a prenatal screening algorithm. Considering tests other than those classified as probable prenatal screens, the female to male ratio of HIV screening tests conducted at CDHA laboratory was close to one to one (1.2:1, 54.8 versus 45.2%).

The 25-29 year age group accounted for 20% of HIV screening tests at the CDHA laboratory, and was also the age group that accounted for the highest percentage of tests when probable prenatal tests were excluded (19% of tests). The age distribution of HIV screening tests followed the same pattern for males and females, with a peak in testing in those aged 20-34 years, and with tests in that age group accounting for 53% of all tests. The demographics of anonymous tests are collected separately by Health Promotion and Protection (HPP). The age and sex distribution of anonymous testers follows a similar pattern to that shown in Figure 1 for non-anonymous tests conducted at CDHA laboratory.

Information about anonymous HIV tests was provided by the Halifax Sexual Health Centre as a non-representative sample of those who are accessing anonymous testing and counselling services as they are currently available in Nova Scotia. The total number of anonymous HIV tests from April 2006 to March 2007 was 522, with 502 the following year, 374 the year after that, and 419 to the end of March 2010. The age group tested most frequently was consistently 20 to 29 year olds (accounting for over 1/3 of tests). The tests were almost evenly split between men and women, with women representing a slight majority of those tested for the first three years, and men representing the majority in the most recent year. Table 1 shows the distribution of anonymous tests conducted at the Halifax Sexual Health Centre by sex and the year they were tested.

**Table 1:** Sex (Self-Identified) of Anonymous Tests by Year, 2006-2010^2^

SEX (SELF-IDENTIFIED)	NUMBER OF TESTS PER YEAR			
	
	*April 06 to March 07*	*April 07 to March 08*	*April 08 to March 09*	*April 09 to March 10*
***Female***	284	297	188	179

***Male***	234	205	186	240

***Not Identified***	4	0	n/a	n/a

**Figure 2:**
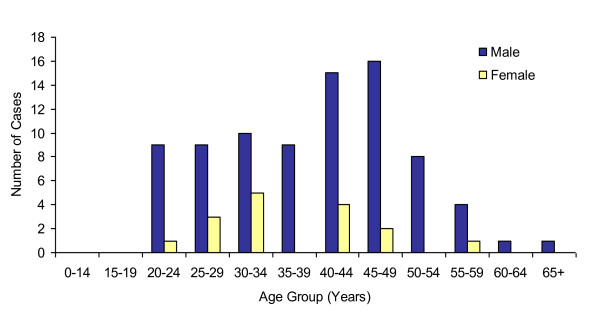
**Newly Diagnosed HIV Cases by Sex and Age Group, Nova Scotia, 2005-2009**.

In contrast to the HIV screening tests, most of the recently reported HIV cases were male (Figure 2). Between 2005 and 2009, males accounted for 83.7% of all newly diagnosed and reported HIV cases. Cases reported between 2005 and 2009 typically followed an older age distribution compared to the HIV screening test distribution (Figures 1 and 2). There was a peak in the number of reported HIV cases in the 40 to 44 and 45 to 49 year age groups, which accounted for 19.4% and 18.4% of all cases, respectively. Additionally, 55.1% of all HIV cases were reported for those 35 to 49 years of age. It appears that newly reported female cases followed a younger age distribution compared to the male cases; however, female case numbers were low.

#### B) HIV Screening Tests: Gender and Setting of Test

Requisitions for HIV screening tests were submitted from 1,081 physicians in Nova Scotia over the time period from April 16^th ^2009 - April 15^th ^2010. The majority of specimens were initiated from physician offices in CDHA (Table 2). Twenty percent of specimens were submitted from outside of CDHA. Nearly 5% of tests were from anonymous testing sites over the time period. Smaller proportions were for specimens collected from individuals in the Queen Elizabeth II (QEII) Emergency Room, QEII inpatients, and QEII outpatients in Halifax.

**Table 2:** Number and Percent of HIV Screening Tests Performed by CDHA Laboratory by Test Setting, Nova Scotia, April 16^th ^2009 - April 15^th ^2010

	n	%
CDHA		
Physician, nominal	9939	64.0
Anonymous Testing Site	730	4.7
Physician, non-nominal	393	2.5
QEII Emergency Room	254	1.6
QEII Inpatient	468	3.0
QEII Outpatient	565	3.6
Outside CHDA	3169	20.4

TOTAL TESTS AT CHDA	15518	

**Figure 3:**
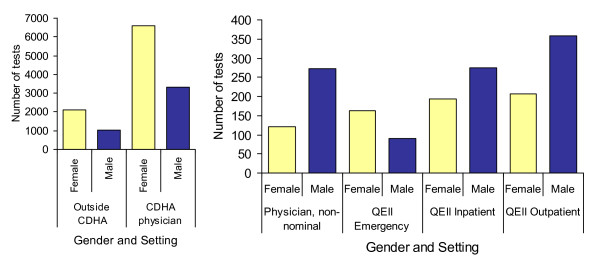
**Number of HIV Screening Tests by Sex and Test Setting, CDHA Laboratory, April 16^th ^2009 - April 15^th ^2010**.

While female HIV screening tests outnumber males tests requisitioned in the physician office setting, more screening tests are conducted on male specimens non-nominally, from QEII inpatients, and from QEII outpatients (Figure 3). Females had higher numbers of tests than males in the QEII Emergency Room (Figure 3).

### In-depth Interview Data

A number of issues relating to gender emerged through the course of the individual, semi-structured in-depth interviews with the 50 adult participants (30 women, 19 men, 1 trans-gendered person). These findings should be viewed as key points that are intended to bring greater awareness to the gender-specific issues related to HIV testing and counselling in Nova Scotia that men and women experience, including the perception and experience of risk factors, the willingness to go for testing, and perceptions of sexual health in general.

The major themes that emerged pertaining to gendered expectations, attitudes and behaviours regarding HIV testing and counselling in Nova Scotia included: the experience and perception of pre-natal screening; the perception of Pap tests and general sexual health services as routine; the availability of accessible testing and counselling services; the experience and perception of stigma and discrimination; and the relationship status and/or perceptions of monogamy as it relates to the decision to be tested.

Based on the interview data, a number of female participants suggested that an HIV test would seem more accessible to them if it was routinized, similarly to prenatal and regular Pap testing that is promoted for women. The availability of accessible HIV testing services encompassed issues of comfort with services, and intersection of gender with other determinants of health, such as ethno-racial and cultural identities. Experiences and perceptions of stigma and discrimination in regards to HIV testing equity were discussed by several participants in regards to their gender, sexuality and sexual identity. Finally, perceptions about sexual relationships and monogamy were also described in terms of gendered norms and expectations regarding HIV testing and counselling.

#### A) Routine HIV Screening

Several participants interviewed had been tested for HIV while they were pregnant. This was generally discussed as a routine process, and this service was routinely offered, as described in the following quotation:

*"I guess they just wanted me to test, 'cause I guess you can get infections and stuff when you're pregnant and they just wanted to check me out and see if I had any STDs..." *(Female, 20s, Aboriginal, High School, HRM, Tested)

In some instances, pregnancy was the main reason participants were tested or considered being tested for HIV. One participant suggested this type of testing was easier for her, and she felt for pregnant women in general, because HIV testing during pregnancy is considered to be routine and normalized as part of the prenatal care offered.

*"For me the testing was done at the time when I was pregnant, so I'm thinking for women who are expecting it's easier for them because it is a test that the doctor usually asks do you want... and I think it happens anyway when you're pregnant." *(Female, 30s, African Nova Scotian, College/University, HRM, Not Tested)

A male participant commented on the fact that HIV testing is routinely, but also exclusively, being offered to women who are pregnant or are planning on becoming pregnant. This is an important gender-based issue in that it may prevent many heterosexual men from coming forward for testing:

*"HIV is not being suggested by health care providers unless you are a woman and you are either expecting a baby or interested in becoming pregnant..." *(Male, 50s, Caucasian, College/University, Outside HRM, Tested prior to past year)

The relationship between HIV testing and general sexual health, Pap tests in particular, also became apparent in conversation with the participants. In a number of instances, female participants mentioned the idea that HIV testing could be normalized, in the way that they perceived Pap tests to be promoted routinely for sexually-active women. The following participants' comments summarize some of the perceptions about HIV testing and the participants' ideas about routinizing this test:

*"I haven't been [tested for HIV] actually. And I know it's probably something everyone should have done, kind of like a woman should always have a Pap smear kind of thing..." *(Female, Teens, Caucasian, Outside HRM, In High School, Not Tested)

*"It [HIV testing] is almost like a Pap test; you have to get it done every year. So I think that everybody should have to get it done every year." *(Female, 30s, Aboriginal, HRM, College/University, Tested prior to past year)

*"For women anyway, maybe it [HIV testing] could be a mandatory part when you go through your yearly check-up, for your Pap test, or maybe it could be a mandatory test that is run when they send your Pap test..." *(Female, 20s, Caucasian, Outside HRM, College/University, Not Tested)

*"I guess making it [HIV testing] more mainstream, like in the same way that people are encouraged... to get their Pap smears at 18 or when you become sexually active. You have your HIV test, you're sexually active, or this age. It should just become a routine part of your health care." *(Female, 20s, Caucasian, College/University, HRM, Not Tested)

Some participants suggested that HIV testing is actually more stigmatized because it is such a specialized test that is set aside from comparably routine sexual health services, such as a Pap test. This sentiment is highlighted in the following:

*"You can't just get it [HIV testing] when you get your regular Pap or anything. It's a very specialized test... HIV is so specific like you get tested for HIV/AIDS and it's done, like drawing blood, it's in its own little category. It's easier to be stigmatized 'cause you can't just say oh I just got tested for STIs and that's all encompassing." *(Female, 20s, Caucasian, College/University, HRM, Not Tested)

Alternatively, another participant suggested that she was more comfortable getting what she perceived to be routine sexual health services, and did not want to be tested for HIV when it was brought up by her health care practitioner:

*"I had gone for a Pap test and I wanted to get some blood work done for other things, I think it was an iron thing and she asked me had I ever gone for an HIV test before and I said no I hadn't... I just wanted to go for my normal Pap test and get my blood work done and go home." *(Female, Teens, Caucasian, HRM, Some High School, Not Tested)

Suggestions about ways to make HIV testing more routine, both in relation to prenatal HIV screening and general sexual health Pap tests, was discussed by many female participants when they were asked about their perceptions about HIV testing. HIV testing was perceived to be routine during prenatal screening, and participants generally promoted the idea that HIV testing would be more acceptable if it was routinely offered similarly to Pap tests.

#### B) Stigma and Discrimination

Stigma and discrimination also emerged as significant to many participants in relation to health equity and HIV testing. Stigma has been defined as an association with socially-unacceptable behaviours resulting in discrimination or social exclusions derived from this association [22]. The stigma and discrimination associated specifically with being tested for HIV was discussed by several participants, particularly in terms of how this intersected with sexuality or ethno-racial identity. One participant commented on the association between AIDS, male homosexuality, and homophobia:

*"I know that there are people out there that automatically assume if you have AIDS that it has to do with homosexuality, that there are people who are homophobic..." *(Female, 60s, African-Nova Scotian, Some College/University, Outside HRM, Not Tested)

One participant discussed that having HIV would compound the stigma and discrimination she already feels based upon her ethno-racial identity.

*"Even though you cannot have it [HIV] by touching me or by sitting beside me, they will run from you... already because you are Black, people don't really associate enough with you anyway." *(Female, 50s, African-Nova Scotian Immigrant, Master's, HRM, Tested prior to past year)

Another participant suggested that stigma and discrimination were the biggest barriers to accessing HIV testing, in specific reference to being gay or transgendered.

*"I think that's one of the big problem is that a lot of people being gay or transgendered or being involved in risky behavior, they don't have the support mechanism, they don't want the stigma or discrimination attached to them." *(Trans, 30s, Caucasian, Some College/University, Outside HRM, Not Tested)

One participant suggested that she and several of her friends who had also immigrated to Canada would prefer to access sexual health services at hospitals, where the reason for the visit would be obscured, given that they did not feel comfortable accessing sexual health services:

*"As far as taking an immigrant woman to get testing, even if the doctor has 100 other things ticked off, she knows everybody knows that this is what she's coming in for. It's a mental state and there's not a whole lot you can do about it other than holding her hand..." *(Female, 50s, African-Nova Scotian Immigrant, Master's, HRM, Tested)

The issues of stigma and discrimination in terms of health equity in HIV testing was shown to influence individual's perceptions and experiences in terms of how gender intersects with ethno-racial identity, sexuality and sexual health-seeking behaviours.

#### C) Sexual Behaviours and Relationships

Perceptions and experiences surrounding sexual behaviours and relationships were prominently discussed amongst the participants in reference to how their sexual activity might be perceived, and also how they perceive their risk of contracting HIV within the content of a monogamous relationship. One participant discussed the link between testing for HIV and the perception of being considered promiscuous:

*"I couldn't go around telling everybody you know I went for an AIDS test or HIV test... cause they'd say oh my god that lady she's promiscuous you know..." *(Female, 50s, Aboriginal, HRM, Tested)

Some participants in monogamous relationships, particularly those who were married or who had been together for many years, did not feel they were at risk for HIV, as suggested in the following quotations:

*"Don't get me wrong; if I had any suspicion that something like that was going on, I would be tested. But personally, like I said, I strongly believe that I'm in a monogamous relationship..." *(Interview 2 - Female, 30, Caucasian, College, Outside HRM, Not Tested)

*"It's not an issue for me cause I've been married for twenty-five years so... no like I say not for me, we just celebrated our 25^th ^wedding anniversary so..." *(Female, 40s, Caucasian, College/University, Outside HRM, Not Tested)

*"I'm not at any risk factors. I'm in a loving relationship so... no I haven't put myself at risk, so why would I want to do that? I get tested for everything, but not even a remote thought about HIV testing." *(Male, 60s, Caucasian, College/University, HRM, Not Tested)

On the other hand, several participants commented on the importance of being tested for HIV, despite being in a monogamous relationship, although often these relationships were not long-term:

*"There's no guarantee that the other person that you're with is not doing something. I mean, you can't be there 24/7 to watch them... nobody else is going to look out for you, so you've got to look out for yourself, to take care of yourself." *(Female, 30s, Aboriginal, College/University, HRM, Tested prior to past year)

*"I've been with him for [several months], but when I met him he was a stranger to me... he told me he wasn't with anyone... he's a pretty nice guy, but you can never be sure." *(Female, 50s, Aboriginal, College/University, HRM, Tested)

Other participants mentioned that a main reason for seeking testing was their sexual partner's desire for them to be tested. Several heterosexual male participants noted that had their female sexual partners not asked for them to get tested, they would not have accessed HIV testing.

*"I think it was more my girlfriend that wanted me to be tested more than my doctor. I think it was like my girlfriend's decision, my girlfriend's decision so..." *(Male, teens, Caucasian, Some high school, HRM, Tested)

Another participant stated that he had been tested for STIs only after his female partner asked him to be tested. The following quote speaks to the gender normative help-seeking behaviors for females which are largely absent for males. When asked what might encourage him to be tested for HIV, he said:

*"I don't know... nothing. Unless I had a spouse or something and she made me..." *(Male, teens, Aboriginal, Some high school, HRM, Not tested)

Other participants still suggested that they were tested or would consider getting tested for HIV prior to getting married or starting a new relationship:

*"I told her, let's both go to the hospital and let's get checked... I said I don't want to have to be accused of messing around. Like condoms, what are they on you for?" *(Male, 30s, Aboriginal, HRM, Tested prior to past year)

*"As a young man... we do have risky behaviors, and sometimes you want [the HIV] test to give you direction to your life... Maybe if you want to marry or something like that." *(Male, 30s, African Nova Scotian Immigrant, Master's, HRM, Tested prior to past year)

In summary, perceptions about monogamy and gendered risk-taking within relationships, including risks associated with the gender-based sexual double standard for males, were shown to be wide-ranging yet significant amongst many participants. Additionally, perceptions of what was considered to be acceptable or unacceptable sexual practices, including perceptions linked to HIV risk and help-seeking behaviours, were also embedded in their gender-based explanations for why they would or would not consider being tested for HIV.

## Discussion

The findings from this research study indicate that there are a variety of gendered implications for HIV testing and counselling in Nova Scotia. Based on both the surveillance and interview data, in terms of who is being testing and who is not, as well as the challenges and facilitators for testing, a number of health equity issues are noteworthy and may have implications for revising the current policy and programming approaches to HIV counselling and testing across the province of Nova Scotia. For example, the quantitative data indicate that approximately half of the women tested for HIV between April 16^th ^2009 and April 15^th ^2010 were tested as part of routine prenatal screening. The interview data of participants' experiences with prenatal testing in Nova Scotia were described as fairly routine. In some instances, routine prenatal testing was described as the only reason why women would consider being tested for HIV and this testing decision was not made with their male sexual partners but rather in relation to their prenatal health care providers' request. To more fully engage heterosexual males in testing, the perceptions of HIV testing as part of the prenatal screening process need to be addressed and reconfigured in a way that is inclusive of this population. It is noteworthy that men who have sex with men are more likely to be tested than heterosexual men, and this in turn has implications for heterosexual women and HIV prevention.

Currently, Nova Scotia's HIV/AIDS provincial policy mandates a routine offer of HIV testing for pregnant women, although the focus on pregnant women misses a large portion of the populations in Nova Scotia who do not regard HIV testing as a health concern, including heterosexual men. Additionally, prenatal screening guidelines in Nova Scotia do not call for testing of sexual partners of pregnant women. While the CDC promotes the idea that opt-out HIV testing should become a routine part of medical treatment in all health-care settings for those aged 13-64 unless the prevalence of undocumented HIV infection is less than 1 in 1000 [23], this approach may further impact the help-seeking behaviours of more socially marginalized populations in Nova Scotia. While it is clear that routine testing has the potential to decrease the stigma associated with HIV testing (as shown by the perceptions about prenatal and Pap screening), the prevalence of HIV in Nova Scotia is relatively low, and may not be cost effective if we used criteria similar to CDC. Additionally, focusing on routine screening for pregnant women has the potential to overlook other populations potentially at risk and less likely to be tested for HIV. In other words, although the routine offer of HIV screening has the potential to normalize HIV testing, research has shown HIV is not a pressing concern for many women [24]. Clearly, gearing HIV testing services specifically towards pregnant women has the potential to downplay the need for comprehensive and equitable sexual health approaches for heterosexual men and also women who are not pregnant or outside typical reproductive age parameters [25].

The surveillance data from this study suggests that most participants access HIV testing through a family doctor or a clinic, not through anonymous testing sites, which may be due to the fact that these sites are limited throughout the province. Although information about who accesses which type of HIV testing service is limited, there were distinct gender differences in where certain types of testing occurred; female HIV screening tests outnumber males tests requisitioned in the physician office setting, although more screening tests were conducted on male specimens non-nominally, from QEII inpatients, and from QEII outpatients. Females also had higher numbers of tests than males in the QEII Emergency Room and the reasons for this require further investigation.^1^

The interview data suggest that not all individuals feel comfortable accessing sexual health services in the same way, and there is a need to provide diverse sexual health services (in terms of location and method of testing) in order to meet the needs of diverse populations and communities. The need to focus on male-specific testing patterns, particularly diverse populations of heterosexual males, is evidenced by the qualitative findings which suggest that most males are unlikely to seek testing without their female sexual partners' recommendation. This suggests the gendered nature of help-seeking behaviours, including HIV testing behaviours, requires augmented efforts to reach males in an effort to shift the current association of HIV testing as solely a normative part of women's routine sexual and reproductive health screening.

The findings of this study reinforce the notion that the current model of HIV service provision is not equitably providing access to all Nova Scotians. Clearly, one approach will not work with all populations and communities, and these populations and communities must be engaged in finding solutions that work for them [26]. Nova Scotia's policy regarding HIV counselling and testing suggests following a harm-reduction model to help decrease the impact of risk behaviours, and expanding options for HIV testing throughout the province (including anonymous testing and point-of-care rapid testing) [26]. It is important to address the barriers to accessing HIV counselling and testing, particularly for those at highest risk and who may be least likely to accept an offer for HIV testing, by developing and implementing various testing strategies to address gaps and specific barriers to testing for diverse communities and their unique needs. In specific reference to the gendered needs of individuals in Nova Scotia, this will require access to gender-sensitive and appropriate services and enhanced sensitivity among health care providers to the gendered norms, expectations and attitudes that influence both males' and females' unique experiences and perceptions of HIV counselling and testing.

As indicated in the data, the highest identified risk categories for HIV infection in Nova Scotia are men who have sex with men (MSM) and injection drug use (IDU), which reflects national trends [1]. The perception of risk, particularly in regards to sexual activity, was highly relevant to the participants of this study as evidenced in the interview data. In light of the fact that heterosexual transmission continues to increase, it is important to combat stigmatizing, homophobic notions about HIV risk categories in isolation from contexts of risk. Nova Scotia's provincial HIV/AIDS policy promotes education and information sharing as a way to help reduce inequities, stigma and misinformation about HIV [21]. It is also imperative that health care providers and researchers consider measures that enhance human rights protection and feasibility, and support research and policy initiatives that attempt to address gendered HIV testing gaps to ensure equitable provision of programs and services [22]. It is only through a more nuanced and gender-sensitive approach that public health efforts will be able to effectively meet the unique needs of Nova Scotia's diverse communities.

## Conclusions

In this paper, we examined the experiences and perceptions of HIV testing and counselling in Nova Scotia, Canada among a diverse sample of adult males and females. Based on the findings of this study, we argue for a more nuanced approach to gender and other key determinants of health as we develop the next wave of HIV testing policies and approaches. Specifically, questioning why particular populations, such as heterosexual men, do not see themselves at risk for HIV and therefore unlikely to test for HIV [19] or why pregnant women are offered HIV testing and in some cases, without their knowledge is warranted [24]. As national and international HIV testing policies and approaches continue to shift, Nova Scotia's provincial testing strategies need to address the gendered perceptions about the utility of current HIV testing approaches and the gendered expectations of who such testing services are meant to serve. This must be done with an eye to the unique needs of diverse communities in an effort to help address the undiagnosed fraction of the population living with HIV in a gender equitable manner so that those diagnosed can benefit from current advances in HIV treatment, care and support.

## Competing interests

The authors declare that they have no competing interests.

## Authors' contributions

JG conceived of the study, contributed to data sampling, analysis, writing of the final report and developed the concept paper. JF coordinated the study, conducted the qualitative data collection, assisted with data analysis and writing. MPS reviewed the concept paper and edited the final version. TH assisted with quantitative data collection and analysis, and reviewed and commented on the final report. LB provided policy data, contributed to writing and editing the paper. All authors read and approved the final manuscript.
